# Case Report: Hybrid retrograde transcarotid stenting for common carotid artery dissection secondary to acute type A aortic dissection

**DOI:** 10.3389/fsurg.2026.1819444

**Published:** 2026-06-18

**Authors:** Jiacai Zuo, Qi Yang, Yi Yang, Zhaokun Li, Xinyu Zou, Xiaochen Gong, Yufeng Tang

**Affiliations:** 1 Department of Neurology, Mianyang Central Hospital, School of Medicine, University of Electronic Science and Technology of China, Mianyang, China; 2 Department of Rehabilitation Medicine, The Third People’s Hospital of Mianyang, Mianyang, China

**Keywords:** acute type A aortic dissection, cerebral malperfusion, common carotid artery dissection, retrograde transcarotid access, stent reconstruction

## Abstract

**Background:**

Endovascular repair is an established option for symptomatic carotid artery dissection, particularly when hemodynamic compromise or embolic risk persists. In patients undergoing repair for acute type A aortic dissection (ATAAD), conventional transfemoral or transradial access may be prohibitive because of altered arch anatomy after graft reconstruction and stent-grafting. We report a postoperative left common carotid artery (CCA) dissection with focal aneurysmal dilatation and cerebral malperfusion following ATAAD repair. The lesion was treated using a hybrid strategy: direct cervical exposure for controlled retrograde transcarotid access and overlapping stent reconstruction.

**Case description:**

A 48-year-old man presented with acute chest and back pain and was diagnosed with ATAAD. He underwent composite aortic repair including aortic root reconstruction, ascending and arch replacement, and descending aortic stent-grafting. Twenty-six hours postoperatively, he developed severe right-sided hemiparesis in the intensive care unit. Computed tomography angiography (CTA) showed near-occlusion of the mid-left CCA and delayed distal opacification. Given unfavorable transfemoral catheterization, emergent surgical cervical exposure enabled retrograde transcarotid sheath placement and deployment of three overlapping self-expanding stents. Final angiography demonstrated complete reperfusion (modified Thrombolysis in Cerebral Infarction, mTICI, grade 3) without intracranial distal embolization. Neurological recovery was favorable, with sustained stent patency and functional independence at follow-up.

**Conclusion:**

Hybrid open retrograde transcarotid access may be considered a salvage option for postoperative CCA dissection after ATAAD repair when transfemoral access is prohibitive.

## 1 Introduction

Endovascular therapy is an established treatment for acute ischemic stroke, particularly in patients with large-vessel occlusion (1). Acute type A aortic dissection (ATAAD) is accompanied by common carotid artery (CCA) dissection in approximately 30% of cases (2), which may lead to cerebral malperfusion and increased mortality (3). When CCA dissection occurs after ATAAD repair and presents with acute ischemic stroke, conventional transfemoral access can be technically prohibitive because of altered aortic arch anatomy following graft reconstruction and stent-grafting.

In selected patients, transcarotid access can provide a direct and controllable route for endovascular intervention when transfemoral catheterization is unstable or not feasible. However, it is used sparingly given concerns regarding access-site complications and embolic risk, and it is generally reserved for cases with complex vascular anatomy or an unfavorable arch configuration, such as a type III aortic arch (4).

Reports of endovascular treatment using surgical cervical exposure to obtain controlled retrograde transcarotid access remain limited. Here, we describe a hybrid open–endovascular approach consisting of direct carotid exposure, retrograde sheath placement, and overlapping stent reconstruction to treat postoperative CCA dissection after ATAAD repair, resulting in favorable clinical recovery.

## 2 Case description

A 48-year-old man was admitted with sudden-onset chest and back pain lasting 3 h. His medical history included hypertension, gout, and long-term tobacco use. Thoracic computed tomography angiography (CTA) demonstrated ATAAD involving the aortic root and arch, extending into the descending thoracic and proximal abdominal aorta with distal propagation to the level of the renal arteries. The cardiac surgery team performed emergent aortic repair under general anesthesia, including aortic valvuloplasty, aortic root reconstruction, ascending and arch replacement, and descending aortic stent-grafting.

After surgery, the patient was transferred to the intensive care unit (ICU). Twenty-six hours postoperatively, he developed acute right-sided weakness. Neurologic examination showed stupor and anisocoria (left pupil 4 mm, fixed; right pupil 2 mm, sluggishly reactive), with dense right hemiparesis and no motor response to painful stimuli; the right plantar response was extensor (Babinski sign). The National Institutes of Health Stroke Scale (NIHSS) score was 26. Emergency head-and-neck CTA revealed near-occlusion of the left CCA with delayed opacification of the internal carotid artery and its intracranial branches. Concurrent computed tomography perfusion (CTP) demonstrated extensive left-hemispheric hypoperfusion with substantial mismatch, consistent with a large volume of potentially salvageable tissue (Figure 1). The Alberta Stroke Program Early CT Score (ASPECTS) was 9.

**Figure 1:**
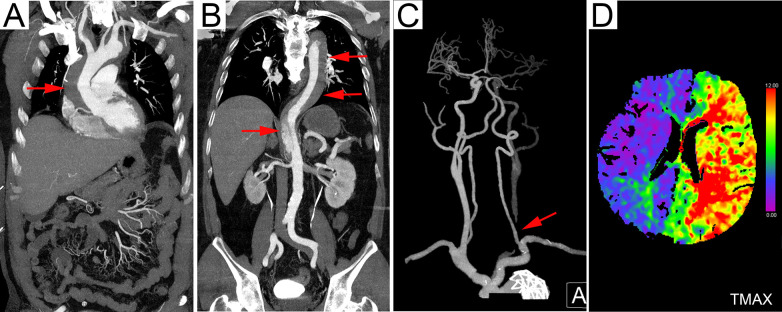
Preoperative and postoperative imaging in a patient undergoing aortic arch repair. **(A,B)** Acute type A aortic dissection extending from the aortic root to the renal arteries (red arrow). **(C)** CTA reconstruction demonstrates subtotal occlusion of the left common carotid artery with delayed contrast opacification of the internal carotid artery and its intracranial branches (red arrow). **(D)** CT perfusion imaging shows extensive hypoperfusion in the left cerebral hemisphere.

## 3 Methods

Cerebral angiography was performed under general anesthesia. Initial transfemoral angiography demonstrated a type III aortic arch. Angiography using a Simmons 2 catheter (Terumo Corporation, Tokyo, Japan) revealed a mid-CCA dissection with focal aneurysmal dilatation, near-occlusion, and impaired distal perfusion (Figure 2A). The initial plan was ultrasound-guided percutaneous retrograde carotid puncture. However, preprocedural ultrasound showed marked ipsilateral jugular venous enlargement with complete overlap of the carotid artery, leaving no safe puncture window.

**Figure 2:**
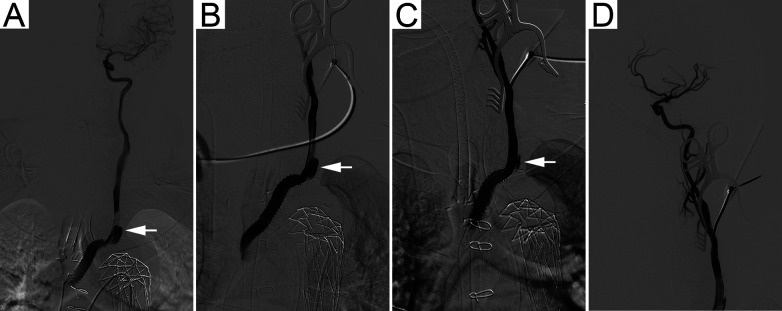
Angiographic images before and after stent reconstruction. **(A)** Preoperative angiography of the left common carotid artery shows an intimal flap originating at the surgical anastomosis and extending distally, with aneurysmal dilatation protruding beyond the vessel wall (white arrow). **(B)** Retrograde angiography through the vascular sheath reveals focal aneurysmal dilatation at the anastomotic site (white arrow). **(C)** Post-stenting angiography demonstrates marked reduction of the focal aneurysmal dilatation and successful recanalization of the vessel (white arrow). **(D)** Final angiography demonstrates successful vessel recanalization with a modified Thrombolysis in Cerebral Infarction (mTICI) grade 3 and no distal intracranial embolization.

Given the unfavorable anatomy, emergent surgical cervical exposure was performed by two vascular surgeons (Figure 3). After careful dissection and direct visualization of the CCA, the puncture site was confirmed by contrast injection, and retrograde access was obtained. A 6-French vascular sheath (Terumo Corporation, Tokyo, Japan) was advanced under fluoroscopic guidance (Figure 2B).

**Figure 3:**
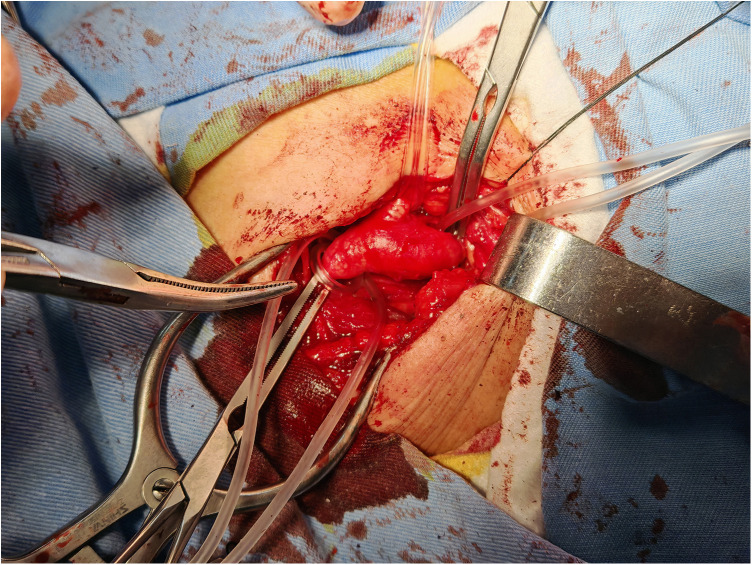
Open cervical exposure of the left carotid bifurcation to enable retrograde puncture under direct visualization. Proximal and distal vessel loops provided temporary vascular control during stent deployment.

A 0.014-inch microguidewire (Boston Scientific, Marlborough, MA, USA) and microcatheter (Micro Therapeutics Inc., dba ev3 Neurovascular, Irvine, CA, USA) were navigated retrogradely across the dissected segment. True-lumen position was verified angiographically, after which the wire was advanced into the descending aorta to provide stable rail support for stent delivery. Because of pronounced curvature at the graft–native vessel junction, an open-cell PRECISE stent (Cordis, Miami Lakes, FL, USA) was selected to optimize wall apposition. Two 8 × 40 mm PRECISE self-expanding stents were deployed with partial overlap under fluoroscopic guidance. Follow-up angiography showed incomplete coverage of the dissected CCA segment, with persistent opacification of the associated aneurysmal dilatation; therefore, a third 8 × 40 mm PRECISE stent was deployed to achieve complete lesion coverage. Final angiography demonstrated reduced opacification of the aneurysmal dilatation, restored antegrade flow with a final modified Thrombolysis in Cerebral Infarction (mTICI) grade of 3, and no evidence of intracranial distal embolization (Figures 2C,D). The sheath was removed, the arteriotomy was repaired, and the neck incision was closed in layers.

## 4 Results

Postoperatively, the patient was monitored in the ICU, with systolic blood pressure maintained at 100–120 mmHg, and received a continuous intravenous infusion of tirofiban (a selective and reversible platelet glycoprotein IIb/IIIa receptor antagonist) for 48 h. Antiplatelet therapy was then transitioned to dual antiplatelet therapy with aspirin (100 mg daily) and clopidogrel (75 mg daily). Atorvastatin was also administered at 20 mg once nightly. Carotid CTA at 24 h confirmed complete stent patency with optimal apposition in the left CCA and no evidence of in-stent thrombosis or malapposition. Brain magnetic resonance imaging (MRI) performed 1 week post-procedure demonstrated a limited infarct burden, with scattered small acute infarcts in the left centrum semiovale, periventricular white matter, and basal ganglia (Figures 4A,B). No access-site complications occurred. The patient was discharged on postoperative day 20 with satisfactory recovery and mild residual right-sided deficits (clumsiness despite 5/5 strength). After discharge, he remained under regular outpatient follow-up and continued daily antiplatelet, statin, and antihypertensive therapy for secondary stroke prevention, with good adherence and tolerability and no documented treatment-related adverse or unanticipated events. At the 18-month follow-up, he had no recurrent cerebrovascular events and only minimal residual weakness confined to the right lower limb, with full independence in activities of daily living (mRS score, 1); from his own perspective, he reported substantial functional improvement. Follow-up CTA demonstrated sustained patency of the left CCA stent without in-stent thrombosis or restenosis (Figures 4C,D).

**Figure 4:**
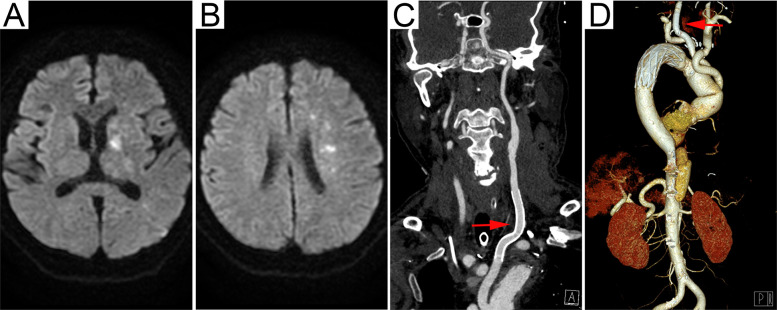
Postoperative cranial MRI and follow-up CTA. **(A,B)** One-week postoperative cranial MRI shows limited acute lacunar infarctions, with scattered small lesions in the left centrum semiovale, lateral periventricular region, and basal ganglia. **(C)** Postoperative follow-up CTA shows the left common carotid artery stent in place and patent, with no evidence of acute in-stent thrombosis (red arrow). **(D)** Follow-up CTA at 18-month post-procedure demonstrates that the stent remains intact and patent (red arrow).

## 5 Discussion

Carotid artery dissection arises from an intimal tear that permits blood to track into the arterial wall, creating an intramural hematoma and separating wall layers. This process can result in luminal stenosis or occlusion, and may produce aneurysmal dilatation when the hematoma extends toward the outer wall (5). In ATAAD, CCA involvement has been reported in up to approximately 30% of cases (2), and cerebral malperfusion with neurological deficits occurs in about 15% (3), both of which are associated with higher postoperative stroke rates and worse neurological outcomes (2, 6). Cerebral malperfusion is an important risk factor for in-hospital mortality; therefore, prompt restoration of cerebral perfusion is critical and has been associated with improved outcomes in ATAAD (7).

Ischemic stroke in the setting of CCA dissection may result from artery-to-artery embolism, flow-limiting stenosis with hemodynamic compromise, or dynamic obstruction by an intimal flap. In ATAAD, additional mechanisms include flap intussusception and compression of the true lumen by the false lumen. Post-repair CCA dissection may reflect fragility of the reconstructed vessel wall and suture-related trauma (8).

Reperfusion strategies span medical and revascularization approaches. Stenting is generally considered for cervical artery dissection with hemodynamic compromise or recurrent ischemic events despite optimal medical therapy (9). Most carotid interventions can be performed via transfemoral access; however, in our patient, prior total arch replacement and severe arch angulation rendered transfemoral and transbrachial catheterization unstable. Under these circumstances, transcarotid access offered the most direct route for revascularization. Although direct carotid puncture carries risks including access-site hematoma, iatrogenic dissection, and sheath kinking (10–12), ultrasound guidance can facilitate true-lumen entry. When percutaneous puncture is not feasible because of jugular venous overlap or an unfavorable puncture window, surgical cervical exposure provides controlled access under direct visualization, enabling precise puncture and reliable hemostasis. Similar hybrid open–endovascular strategies have been reported for acute ischemic stroke, intracranial aneurysms, and severe carotid stenosis when conventional transfemoral access is limited by anatomic or technical constraints (13, 14). While this approach typically requires general anesthesia and may be more time-consuming than percutaneous techniques, workflow can be optimized in a hybrid operating room with high-quality angiography (13, 14).

In our case, the aneurysmal outpouching of the dissected CCA was consistent with dissection-related aneurysmal dilatation. Large or symptomatic aneurysmal dilatation may increase the risk of thromboembolism and progressive enlargement, often prompting endovascular or surgical intervention. Endovascular stent reconstruction remains the most commonly used approach and has been reported as safe and effective in selected patients (15).

Open surgical reconstruction represents an alternative for CCA occlusion or complex lesions, including cervical reimplantation, interposition grafting with autologous or synthetic conduits, or surgical fenestration (7, 16). Bypass options (e.g., subclavian-to-internal carotid or vertebral-to-internal carotid bypass) have also been described (17, 18). However, these procedures are technically demanding and time-consuming, involve greater surgical trauma, and may prolong time to reperfusion in the acute stroke setting.

A prior report by Lin et al. described postoperative right CCA and internal carotid artery occlusion after ATAAD repair, requiring surgical exposure and multiple punctures for deployment of five stents (19). In contrast, our patient had left-sided CCA dissection with focal aneurysmal dilatation. After angiographic identification of the true lumen, stent reconstruction was performed via a single controlled puncture, potentially reducing access-related injury and inadvertent false-lumen entry. Mukherjee et al. reported four patients with Type A aortic dissection complicated by cerebral malperfusion, in whom retrograde carotid stenting restored cerebral perfusion with favorable outcomes (20). This supports the feasibility of this approach in selected patients.

Regarding stent selection, Mukherjee et al. used VICI and Abre venous stents (20), considering that their radial resistive properties may help displace the dissection septum and restore the true lumen. Agarkov et al. also reported carotid stent compression by a false-lumen hematoma at autopsy, highlighting the importance of radial support in this setting (21). However, direct comparative data between venous stents and dedicated carotid stents are lacking. In the present case, open-cell PRECISE carotid stents were selected because of marked vessel tortuosity and local device availability, and were deployed in an overlapping manner to improve wall apposition and increase local metal coverage density. Additionally, in their reported cases (20), revascularization was performed promptly once clinical or radiological evidence of cerebral malperfusion was identified, before established stroke occurred. This suggests that early recognition and timely treatment may be beneficial in selected patients. Because a cerebral protection device could not be used, adjunctive measures—including distal carotid control with a vascular loop during device manipulation—were used to mitigate embolic risk in the absence of balloon-guide catheter support. Together, these cases suggest that hybrid open retrograde transcarotid stenting is a feasible salvage option when conventional transfemoral access is unstable or not feasible.

After revascularization, maintaining systolic blood pressure at 100–120 mmHg, as recommended by current guidelines (22), may help preserve cerebral perfusion while avoiding excessive aortic wall stress. Because carotid stenting was performed soon after aortic surgery, antiplatelet therapy should be individualized by balancing stent thrombosis prevention against postoperative bleeding risk. In our case, tirofiban was used as a bridge to dual antiplatelet therapy, without bleeding complications. Statin therapy may be considered when concomitant atherosclerotic disease, dyslipidemia, or other vascular indications are present.

This report highlights a hybrid open retrograde transcarotid stenting strategy as a salvage approach for postoperative CCA dissection with cerebral malperfusion after aortic repair. Limitations include its single-case nature; further studies are needed to validate long-term efficacy.

## 6 Conclusion

Hybrid open retrograde transcarotid access with endovascular stent reconstruction may be a feasible salvage strategy for postoperative CCA dissection with focal aneurysmal dilatation after ATAAD repair when transfemoral access is not feasible.

## Data Availability

The original contributions presented in the study are included in the article/Supplementary Material, further inquiries can be directed to the corresponding author.
