# Supplementary material for: Case Report: Hybrid retrograde transcarotid stenting for common carotid artery dissection secondary to acute type A aortic dissection
Source: Front Surg. 2026 Jun 18;13:1819444. doi: 10.3389/fsurg.2026.1819444 (PMC13323301; doi:10.3389/fsurg.2026.1819444)
Supplement: Supplementary file 1 [file Table1.docx]

**Supplementary Table- Timeline**

**Case Report:** **Hybrid Retrograde Transcarotid Stenting for Common Carotid Artery Dissection Secondary to Acute Type A Aortic Dissection**

| **Time point** | **Event / Assessment** | **Key findings** | **Management / Outcome** |
| --- | --- | --- | --- |
| Day 0 (Symptom onset → admission; ~3 h) | Presentation and initial imaging | Sudden-onset chest and back pain; thoracic computed tomography angiography (CTA) confirmed acute type A aortic dissection (ATAAD) extending to the renal arteries | Emergent surgical planning |
| Day 0 (same day) | Aortic surgery | ATAAD repair: aortic valvuloplasty; aortic root reconstruction; ascending and arch replacement; descending aortic stent-grafting | Postoperative ICU care |
| Postoperative +26 h | Neurologic deterioration | Acute right-sided hemiparesis; National Institutes of Health Stroke Scale (NIHSS) 26 | Emergent neuroimaging and angiography decision |
| Postoperative +26 h (same episode) | Head-and-neck CTA + computed tomography perfusion (CTP) | Near-occlusion of left common carotid artery (CCA) with delayed distal opacification; extensive hypoperfusion with substantial mismatch; Alberta Stroke Program Early CT Score (ASPECTS) 9 | Proceeded to angiography and revascularization |
| Postoperative +26 h (procedure day) | Digital subtraction angiography (DSA) | Type III aortic arch; mid-CCA dissection with focal aneurysmal dilatation and impaired distal perfusion | Planned transcarotid access due to unstable/prohibitive transfemoral route |
| Postoperative +26 h (procedure) | Hybrid open retrograde transcarotid stenting | Jugular venous overlap precluded percutaneous puncture | Open cervical exposure → retrograde CCA puncture → sheath placement → deployment of three overlapping stents; final modified Thrombolysis in Cerebral Infarction (mTICI) 3; no intracranial distal embolization |
| Post-procedure 0–48 h | Antithrombotic therapy | — | Intravenous tirofiban infusion for 48 h, then dual antiplatelet therapy (aspirin + clopidogrel) |
| Post-procedure +24 h | Carotid CTA | Complete stent patency; optimal apposition; no in-stent thrombosis/malapposition | Continued medical management |
| Post-procedure +1 week | Brain magnetic resonance imaging (MRI) | Scattered small acute infarcts; limited infarct burden | Rehabilitation and follow-up planning |
| Postoperative day 20 | Discharge | Mild residual right-sided deficits | Discharged with antiplatelet therapy and follow-up plan |
| 18 months (follow-up) | Clinical + imaging follow-up | No recurrent cerebrovascular events; independent activities of daily living; modified Rankin Scale (mRS) 1; CTA showed sustained stent patency without restenosis | Favorable long-term outcome |
